# Phenotypic and genetic divergence within a single whitefish form – detecting the potential for future divergence

**DOI:** 10.1111/eva.12087

**Published:** 2013-09-10

**Authors:** Philipp Emanuel Hirsch, Reiner Eckmann, Claus Oppelt, Jasminca Behrmann-Godel

**Affiliations:** 1 Program Man-Society-Environment, University of BaselBasel, Switzerland; 2 Limnological Institute, University of KonstanzKonstanz, Germany

**Keywords:** adaptive radiation, resource polymorphisms, respeciation, sympatric speciation

## Abstract

Human-induced nutrient input can change the selection regime and lead to the loss of biodiversity. For example, eutrophication caused speciation reversal in polymorphic whitefish populations through a flattening of littoral–pelagic selection gradients. We investigated the current state of phenotypic and genetic diversity in whitefish (*Coregonus macrophthalmus)* in a newly restored lake whose nutrient load has returned to pre-eutrophication levels and found that whitefish spawning at different depths varied phenotypically and genetically: individuals spawning at shallower depth had fewer gill rakers, faster growth, and a morphology adapted to benthic feeding, and they showed higher degrees of diet specialization than deeper spawning individuals. Microsatellite analyses complemented the phenotype analyses by demonstrating reproductive isolation along different spawning depths. Our results indicate that whitefish still retain or currently regain phenotypic and genetic diversity, which was lost during eutrophication. Hence, the population documented here has a potential for future divergence because natural selection can target phenotypes specialized along re-established littoral–pelagic selection gradients. The biodiversity, however, will have better chances to return if managers acknowledge the evolutionary potential within the local whitefish and adapt fishing and stocking measures.

## Introduction

Understanding the origins of biodiversity is a fundamental and long-standing motivation for biologists. Disciplines such as ecology, evolutionary biology, and conservation biology are unified in their efforts to analyze the processes that generate and maintain diversity (Hendry et al. 2011; Matthews et al. 2013). In recent years, researchers have increasingly worked toward conserving biodiversity in pristine ecosystems and even to re-instate biodiversity after it was lost due to anthropogenic impacts (Hendry et al. 2011; Matthews et al. 2013). The prospect to bring back biodiversity in natural ecosystems requires a detailed understanding of the evolutionary principles that allow for the diversification of lineages. For a successful ‘re-speciation’ (*in sensu* McKinnon and Taylor 2011), however, two fundamental conditions must be met. First, the selection regime that historically allowed for diversification along environmental gradients needs to be restored (e.g., habitat restoration, re-installation of ecotones: (Smith et al. 1996; Collyer et al. 2007). Second, and even more important from an evolutionary perspective, there needs to be a source of heritable variation upon which selection can act after selection gradients have been restored.

Freshwater fish in lake ecosystems are among the best-studied model systems for the study of factors that promote or constrain the diversification of lineages (Schluter 1990; Bernatchez et al. 2010). The existence of benthic and pelagic habitats in lakes can create a disruptive selection regime favouring extreme phenotypes: a pelagic phenotype, feeding on zooplankton, and a littoral phenotype specialized for benthic feeding (Robinson and Wilson 2009; Svanbäck and Eklöv 2003). Mathematical models and empirical data clearly highlight the significance of bimodal resources for phenotype diversification (Thibert-Plante and Hendry 2002; Hirsch et al. 2013). Thus, only in an environment that features distinct resources across an environmental gradient, disruptive selection will favor extreme phenotypes, while intermediate phenotypes lie at a fitness minimum and are disfavored.

**Figure 1:**
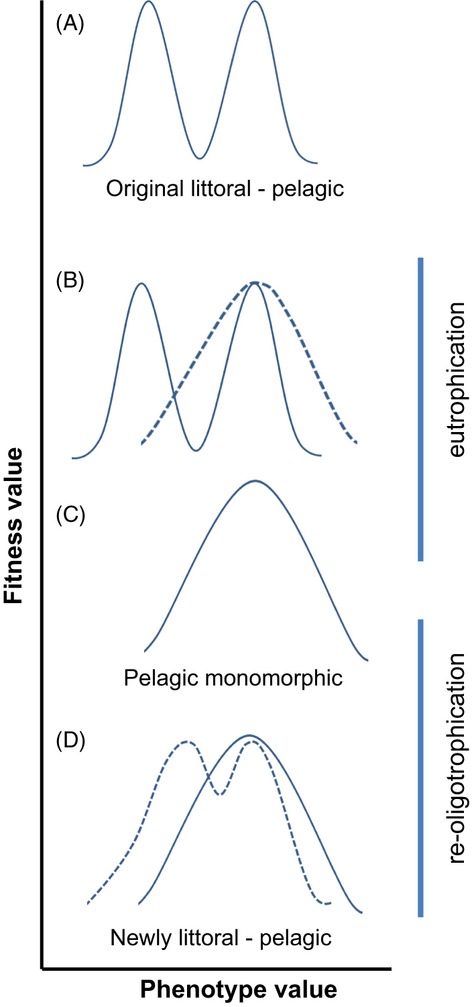
Possible development of the selection regime expressed as fitness versus phenotype value during eutrophication and re-oligotrophication. The solid lines indicate the fitness–phenotype relationship, and the dashed lines indicate a change in the relationship (A) in a pristine lake there were two adaptive peaks for phenotypes specialized on either pelagic or benthic resources and a fitness minimum for intermediate phenotypes. (B) During the process of eutrophication, the benthic resources would be ‘disproportionally affected’ (Vonlanthen et al. 1999) by the change in nutrient load and benthic feeding phenotypes will be less favored by selection. Pelagic-feeding phenotypes in contrast would experience little or no change of their selection landscape because the pelagic resources increased in density (see the zooplankton data from Fig. 2, this article). (C) The loss of the bimodality of the resource distribution would lead to the two adaptive peaks converging, presumable somewhere near the pelagic optimum. (D) Following re-oligotrophication, the bimodality of resources would be restored (see the zooplankton data from Fig. 2, this article), and hence, the adaptive peaks and the fitness minimum re-emerge where they had been originally. Natural selection can now again act to favor specialized benthic feeding phenotypes if those are still present in the population.

It is widely acknowledged that the magnitude of diversification within a polymorphic population can vary substantially across ecosystems and historical timescales (Siwertsson et al. 2008; Bartels et al. 2012; Hirsch et al. 2013). Recently, there is an increasing appreciation of the role human-induced environmental change can play in changing the degrees of diversification (Hendry et al. 2006). For example, eutrophication (a lake's response to human-induced nutrient input) has been implicated in the loss of fish diversity through speciation reversal (Seehausen 2000; Vonlanthen et al. 1999). The underlying mechanisms of how exactly eutrophication leads to speciation reversal are untested, but one plausible explanation involves the loss of the typical bimodality of benthic and pelagic resources in eutrophied lakes. With resources not containing profitable rewards at two distinct points of the resource axis, the fitness gain of specializing on distinct benthic resources are lost upon eutrophication (Vonlanthen et al. 1999) (Fig. 1). Ecological theory predicts that if the ecological opportunity of specializing on underutilized resources does not provide a fitness advantage relative to unspecialized competitors, then selection becomes stabilizing favoring a unimodal distribution of phenotypes within a population (Seehausen 2000; Seehausen et al. 2006). Most eutrophied lakes are characterized by dramatic increases in the biomass of zooplankton accompanied by losses in the benthic invertebrate biomass (Jeppesen et al. 1998; Anderson et al. 2005; Vonlanthen et al. 1999). Fewer benthic resources in concert with an oversupply of zooplankton are assumed to decrease the rewards individuals will receive from specializing on benthic resources relative to their pelagic conspecifics because competition is relaxed (Anderson et al. 2005; Vonlanthen et al. 1999) (Fig 1). Hence, the fitness gain of specializing on distinct benthic resources is lost upon eutrophication (Vonlanthen et al. 1999) (Fig. 1). This mechanism has most likely caused a loss of traits connected to benthic feeding in whitefish across twelve prealpine lakes in Central Europe (Vonlanthen et al. 1999), and as a consequence, previously distinct benthic and pelagic whitefish species became phenotypically indistinct.

Ecological theory posits further that reproductive isolation is favored if the trophic traits of a phenotype are connected to reproductive traits (Schluter 1996). Accordingly, in whitefish where specialized phenotypes with distinct feeding habitats aggregate in spatially and/or temporally separated clusters during spawning, genetic diversification can follow as a byproduct of trophic separation. The loss of trophic separation among whitefish forms therefore entails a loss of genetic separation: As more and more individuals share the same resource they also hybridize during spawning which leads to the genetic collapse of whitefish forms into a monomorphic hybrid swarm (Seehausen 2000; Seehausen et al. 2006).

Human impacts on ecosystems and selection regimes can, however, be mitigated or even reversed. For example, re-oligotrophication (a lake's response to decreasing nutrient inputs) has been reported from lakes in industrialized countries worldwide (Anderson et al. 2005). Once pristine environmental conditions have been restored, also the bimodality of resources and the resource competition that can cause diversification can re-emerge. For the lost biodiversity to re-appear, however, there needs to be a phenotypic diversity on which natural selection can act upon to favor individuals specifically adapted to the newly bimodal resources. We predict that specific investigations into phenotypic and genetic divergence within a local population should reveal such remaining easy-to-overlook biodiversity and will then allow to plan further steps toward regaining whitefish biodiversity (McKinnon and Taylor 2011).

We further predict that detecting an easy-to-overlook biodiversity in a recently restored ecosystem comes with management implications. For example, during the early phases of the diversification into a pelagic and a littoral form, the latter will be numerically inferior to the former. Hence, the littoral form would need special protection, for example via catch restrictions or adaptations in stocking practices, to support the re-emergence of the littoral phenotype. To investigate whether such easy-to-overlook biodiversity could be detected in a recently restored lake ecosystem, we used a whitefish species (*Coregonus marcophthalamus*; ‘gangfisch’) endemic to Lake Constance as a model species. The lake lost its historical whitefish species diversity during eutrophication, but its nutrient load has now returned to pre-eutrophication levels. We hypothesized that a local gangfisch population shows subtle within-population diversification into distinct benthic and pelagic phenotypes.

## Study system and methods

### The history of whitefish diversity in Lake Constance

Before eutrophication during the second half of the 20th century, the species diversity in Lake Constance reflected the frequently observed pattern of diversification into two benthic and two pelagic species (Ostbye et al. 1978; Ostbye et al. 2005b). The two benthic species were the kilch: *C. gutturosus* and the sandfelchen: *C. arenicolus*. The two pelagic species were the blaufelchen: *C. wartmanni* and the gangfisch: *C. macrophthalmus*. All four species regularly occurred in commercial fisheries catches (Nümann 2012). All species were clearly differentiated based on the location of their spawning areas and their ecology. The kilch was a small, benthic feeding deep-water species spawning between July and November that occurred in depths over 70 m. The big benthic feeding sandfelchen occupied the shallow littoral zone spawning in late January in very shallow water near the shoreline. The gangfisch was a pelagic, mainly zooplankton feeding species that occupied the near-shore areas where it spawned between December and January close to the bottom. The blaufelchen was the pelagic open-water plankton-feeding species that spawned at the beginning of December near the surface offshore in the middle of the lake. Besides the different spawning locations and seasons, the number of gill rakers was used to distinguish the sparsely rakered benthic feeders kilch and sandfelchen from the densely rakered pelagic feeders gangfisch and blaufelchen (Table 1).

**Table 1:** Historical and contemporary gill raker numbers (GRN) of whitefish under oligotrophic (1963/67), eutrophic (1990), and re-oligotrophic (2009/10) conditions of Lake Constance

Species	Year	N	GRN (mean)	Year	N	GRN (mean)	Year	N	GRN (mean)
Kilch									
* C. gutturosus*	1967*	112	17–25 (21)	1990	–	No record	2009	–	No record
Sandfelchen									
* C. areniocolus*	1967*	25	25–29 (–)	1990	–	No record	2009	–	No record
Blaufelchen									
* C. wartmanni*	1963*	534	30–40 (36)	1990†	40	31–41(37)	2009/10‡	61	31–36 (33)
Gangfisch									
* C. macrophthalmus*	1967*>	115	33–43 (38)	1990†	40	30–42 (37)	2009¶	90	23–42 (35)

* Nümann 2012.

† Luczynski et al. 1995.

‡ Rösch FFS personal communication.

¶ This study.

**Figure 2:**
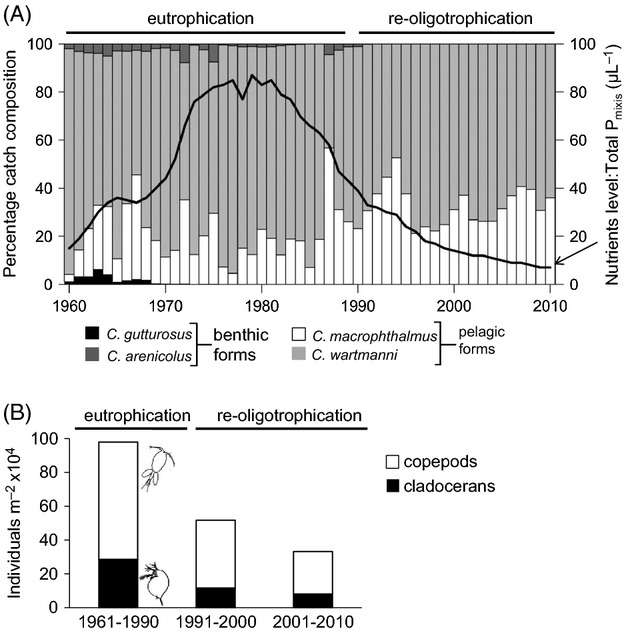
Historical context of the whitefish (*Coregonus* spp.) forms in Upper Lake Constance. (A) After a period of eutrophication, the nutrients (total phosphorous concentration during winter mixis) are today back at pre-eutrophication levels. Catches of whitefish from commercial fisheries indicate that the benthic forms have disappeared following the eutrophication. (B) Zooplankton density decreased since re-oligotrophication. Data show means for the eutrophication period (1961–90) and for the two decades of the re-oligotrophication. Zooplankton data are collection samples of vertical hauls from the middle of Upper Lake Constance (latitude = 47.62°, longitude = 9.39°) down to approximately 100 m depth. Commercial fisheries landing data, data on phosphorous, and zooplankton data courtesy of the International Commission for Fisheries at Lake Constance and the International Commission for Water Protection of Lake Constance (IGKB 2011).

Subsequently, by speciation reversal related to eutrophication in the late 1980s, the lake lost part of this whitefish biodiversity, and only the two pelagic species, the gangfisch and the blaufelchen remained abundant and commercially important during the lake's recent history (Fig 2 A). Before eutrophication, blaufelchen and gangfisch were reproductively isolated and separated in their feeding and spawning grounds (Eckmann and Roesch 1998; Vonlanthen et al. 1999). Both blaufelchen and gangfisch use zooplankton as main food resource. However, historical records indicate that, in addition to zooplankton, gangfisch diets also contained benthic prey items, for example, chironomids (Steinmann 1994). During the eutrophication period, however, both gangfisch and blaufelchen occupied the same offshore open-water feeding grounds and foraged on the eutrophication-fostered zooplankton stock (Eckmann and Roesch 1998; Roesch 1994). The spawning grounds of gangfisch have changed remarkably during the process of eutrophication and re-oligotrophication of the lake. Typically, the spawning grounds of gangfisch reached from shallow areas around 10-m-depth down to 70-m-deep regions of the lake (Steinmann 1994; Wagler 2009; Nümann 2012). During eutrophication, the spawning grounds of gangfisch became restricted to shallower areas between 5 and 20 m depth, (Nümann 2012; Eckmann and Roesch 1998). The best documented change in whitefish diversity due to eutrophication is the contraction of the overall range of gill raker numbers across species in the lake (Table 1) (Vonlanthen et al. 1999).

As expected from the disproportionate impact of eutrophication on the benthic habitats, the two benthic whitefish species were more severely affected by eutrophication. The kilch (*C. gutturosus* Fig 2A) went extinct during eutrophication (Vonlanthen et al. 1999). The sandfelchen was still occasionally caught in commercial catches, but its numbers have not recovered since the dramatic loss during eutrophication (*C. arenicolus* Fig. 2A). Time series of genetic data from scale archives provide further evidence for the genetic admixture of the previously separated species in the lake. Pairwise genetic differentiation between extant species as well as global *F*_ST_ values have dropped during the course of eutrophication (Vonlanthen et al. 1999). Direct comparison of the microsatellite allele distributions indicates interbreeding occurred, at least between the nowadays extinct *C. gutturosus* and the remaining species. Some formerly private *C. gutturosus* alleles can now be found in the other species. Interestingly, most of the former *C. gutturosus* alleles appear in gangfisch (Vonlanthen et al. 1999). The gangfisch of Upper Lake Constance is a particularly suitable model for investigating the present degree of within-species diversification: as stated above, its diet included benthic items prior to eutrophication. Most notably, spawning grounds of whitefish are expanding again to shallower (<5 m) and deeper regions of up to 50 m depth (local fishermen and pers. obs.). In whitefish, benthic and pelagic resource specialists are known to separate in spawning depth, hence providing opportunities for reproductive isolation to follow phenotypic divergence between habitats through by-product speciation (Ostbye et al. 2005a; Vonlanthen et al. 2004).

### Field sampling

Upper Lake Constance has a surface area of 472 km^2^ with a mean depth of 101 m and a maximum depth of 250 m. We caught individuals during spawning season in November 2009 from three depths (2, 25, and 50 m) near the limnological institute of Konstanz University (47.6957° latitude, 9.1942° longitude) where the lake bottom gradually descends to approximately 10 m depth at around 50 m from the shore and then more steeply reaches down to 100 m. At each depth, three gill nets of 32-, 38-, and 44-mm bar mesh were set parallel to the shoreline. Nets were set in the evening and lifted early next morning. From each depth, 30 fish were randomly sampled. The sampling site was chosen at a known spawning location of local gangfisch. Blaufelchen do not spawn in this area, and the use of bottom nets further excluded an accidental catch of pelagic-spawning blaufelchen.

### Scalometry and comparison of growth trajectories

Scales for age determination and size-at-age back calculation were taken from the area covered by the ventral fins. Age was determined from annular marks, and their radii (i.e., the distances between the center of the scale and each annual mark) were measured from digital images. Length-at-age was then back-calculated using our own several years spanning, unpublished database of linear regressions of fish total length, and scale radius as a reference. To test for differences in growth trajectories (i.e., the rate at which the asymptotic body size is reached), we followed Etheridge et al. (2010b) approach for comparing whitefish growth rates; we performed an ancova with the different depths as a categorical predictor to test whether the relationship between age and length (i.e., the slope of the growth curves) differs between depths.

### Trophic morphology and body shape analyses

All analyses were carried out on freshly dead fish, within four hours after catch. To specifically address the feeding morphology of the gangfisch, we counted the number of gill rakers on the first right gill arch. For geometric morphometrics, each individual was photographed with its fins spread and fixed to the surface of a polystyrene bed. We chose 16 landmarks on the left side of each specimen following general guidelines for placement of landmarks (Zelditch et al. 1991) (Fig. S1). Because large filled gonads can influence the body shape in the ventral body regions (Helland et al. 2009), we omitted the commonplace landmark at the pelvic fin from all analyses. To quantify morphological variation in body shape among individuals, we performed multivariate geometric shape analysis. After digitizing the landmarks using TpsDig, we analyzed each landmark's relative position and hence overall variation in body shape using TpsRW (Thin-Plate Spline Relative Warp), (Rohlf 2004) (all Tps-software and information available for download at http://life.bio.sunysb.edu/morph/index.html). TpsRW allowed calculation of the partial warp and uniform scores that denote the differences in body shape among the individuals. Both partial warps and uniform scores were scaled to centroid size as part of a generalized Procrustes analysis (GPA; please refer to Rohlf and Slice (2000) for details of the method). We then analyzed the partial warps and uniform scores using a multivariate discriminant function analysis (DFA) based on the classification of individuals into the different spawning depths. A subsequent canonical variance analysis (CVA) combined all partial warp and uniform scores for each individual into two CVA scores that maximally discriminate between the three depths. The CVA scores were used solely for visualization of the differences in morphology because they represent single values for an individual that are easy to use in software designed to visualize shape differences. For visualization of the body shape differences among depths, we connected the landmarks of two extreme individuals that lie on opposite ends of the morphology spectrum ranging from what the CVA discriminated as the most littoral and the most pelagic individual. Body shape depictions were created using the software TPSregr that regresses the variation in body shape with independent variables such as CVA scores or measures of genetic differentiation such as FCA scores. To test for specific differences in morphology between the different depths, we performed a mancova with all partial warps and uniform scores as response variables and depth as a categorical predictor variable. To account for any level of allometric size variation caused by differences in size at age in gangfisch, we included fish size (log centroid size) as a covariate in our model. To control for a possible artifact of body arching (*cf*. Valentin et al. 2002), we used the function ‘unbent’ as implemented in the software tpsUtil. We therefore first connected the landmarks 1, 6, 9, and 10 with a straight line using the software's graphical interface (Fig. S1). The software then fits a quadratic curve through this line of landmarks and ‘unbends’ the configuration so that the estimated quadratic fit becomes a perfectly horizontal line. The tps-file created by applying this unbent configuration contains the shape information without any bending artifacts were used for all further analyses. All statistics were performed using Statsitica vers. 11 (Statsoft® (Europe), Hamburg, Germany) except for the tps-software (see above), niche metrics, and genetic analyses (see below).

### Stable isotope analyses

Because individuals were caught during spawning time, most specimens had an empty stomach which precluded stomach content analyses. To assess the trophic niches within gangfisch from different depths, we followed Layman et al.'s (2007) approach for assessing the trophic niche of individuals or communities with carbon and nitrogen stable isotopes. The applied niche metrics are based on Leibold's (1995) account of the niche as the ‘trophic role’ of an individual or species. As we were interested in the long-term niche of the gangfisch spawning at different depths, we used the fish scales for N^15^ and C^13^ analyses. Fish scales are a tissue that is continuously synthesized but does not experience biological turnover. Hence, scales are an exceptionally useful tissue to study long-term diet use of whitefish (Rennie et al. 2011). Scales were cleaned from adhering tissue and rinsed with distilled water until all scales were free of any organic residue. After air-drying for 2 days, we removed the center of each scale with a 2-mm-gauge punch. This excluded scale tissue that is associated with the early life stages of whitefish in which all individuals are obligate zooplanktivorous (*cf* Rennie et al. 2011). Based on the age calculations for the specimen, we inferred that the scale excluding the center integrated over an average of 1.98 years (±0.64 SD) of an adult fish's life. Scales were dried at 60°C for 24 h. Each scale was then dorso-ventrally cut in half, and the remaining half was further cut into small (<1 mm) pieces of 0.8–1.2 mg weight. The fine-cut scale pieces were subsequently weighed and wrapped into 6 × 4 mm tin capsules for elemental analysis (Elemental Microanalysis Ltd, Okehampton, UK). Samples were then commissioned to the Stable Isotope Facility of the University of California in Davis and analyzed by a continuous-flow isotope ratio mass spectrometer (PDZ Europa 20-20). The results are expressed using the delta (δ) notation in ‰ as δ = (*R*_sample_/*R*_standard_ − 1) × 1000, where *R* = ^13^C/^12^C and ^15^N/^14^N, and Pee Dee belemnite and atmospheric nitrogen was used as a standard.

### Trophic niche metrics

#### Niche width and niche segregation

Using the signatures of heavy stable isotopes of nitrogen (δ N^15^) and carbon (δ C^13^) as long-term integrators of an individual's diet, one can describe an individual's position in the biplot of a two-dimensional niche space (for a detailed theoretical and methodological background of the metrics applied, refer to Layman et al. (2007). The first metric we applied is the total niche area (TA). It encompasses the total amount of niche space occupied by a set of individuals and is measured as the convex hull area (the smallest possible polygon comprising all data points in a two-dimensional space, Fig. S3). The TA can be strongly influenced by points with extreme positions along either the δ C^13^ or δ N^15^ axis. As a more robust measure of total niche area, Jackson et al. (2011) developed a congruent measure of niche space based on the standard ellipse area (SEA) that is more robust against outliers (Fig. S4).

Following Quevedo et al. (2003), we calculated the overlaps of the convex hulls and additionally of the standard ellipse areas as measures of niche segregation between gangfisch spawning at different depths.

#### Trophic diversity and evenness

As a measure of trophic diversity, Layman et al. (2007) put forward the mean distance to centroid (CD). The CD describes how far individuals within one population are scattered around the population niche mean. We used CD as a measure of diet specialization among individuals caught at a certain depth. If all individuals specialize on the same diet items, the CD will become small. A higher degree of individual diet specialization results in a large scatter of individuals around the population mean. CD is calculated as the average Euclidian distance between each individual's point in the biplot and the centroid. The centroid is the point in the biplot that depicts the mean value of all the individuals' δ N^15^ and δ C^13^ values. An added measure of trophic diversity in the form of trophic evenness is the mean nearest neighbor distance (MNND). It describes how far an individual in the population is away from the other individuals, that is, the mean of the Euclidean distances to each individual's nearest neighbor. If all individuals within a population feed on the same diet items, the MNND will be small and the trophic evenness high. Conversely, if the diet items differ greatly between individuals, the MNND is larger. Hence, evenness would be lower at higher values, which appears somewhat counter-intuitive. We therefore provide the MNND-1 as a more intuitive measure. With increasing decimal values, the trophic evenness increases, that is, with the value for trophic evenness approaching 1, more individuals share the same diet items. All metrics are calculated separately for all individuals caught at 2, 25, and 50 m depth. All niche metric analyses were performed using the SIAR package in R (Jackson et al. 2011). Prior to the niche metric analyses, we performed anovas to check for overall differences in nitrogen (δ N^15^) and carbon (δ C^13^) signatures between depths.

### Genetic population differentiation

#### DNA extraction and microsatellite analysis

Fin clips from each fish were preserved in 95% ethanol for later genetic analysis, and DNA was extracted using a standard salt extraction method (Aljanabi and Martinez 1997). Altogether, twelve microsatellites were multiplexed in two batches. The first batch contained the markers Cocl-lav 6, 10 and 68 (Fam-labeled), Cocl-lav 49 and 61 (Hex-labeled), Cisco157 and SSBgIIM (Pet-labeled), the second batch contained BWF2 and Cocl-lav 45 (Fam-labeled), BWF1 and Cocl-lav 18 (Hex-labeled) and Cocl-lav 4 (Pet-labeled) (Patton et al. 2006; Turgeon et al. 2011; Rogers et al. 2006). This or similar sets of markers have been previously used to assess the population structure in other central alpine coregonids (see Vonlanthen et al. 2004 for detailed description of specific conditions for multiplexing of loci). The two batches were fragment analyzed on an ABI3130 genetic analyzer with the Genescan LIZ600 (Applied Biosystems Inc., Foster City, CA, USA) sizing standard. Allele calling was conducted automatically with the Genemapper software and controlled visually.

#### Analysis of genetic diversity

All loci were checked for the presence of null alleles with the software MicroChecker (Van Osterhout et al. 2004). Observed (*H*_O_) and expected (*H*_E_) heterozygosity of samples from the three different depths were calculated using arlequin version 3.5 (Excoffier and Lischer 2010). Deviations from Hardy–Weinberg equilibrium (HWE) were tested using exact tests (Guo and Thompson 1992), for each locus and sample using genepop vers. 4.0. (Raymond and Rousset 2009). We ran 20 batches and used 10 000 dememorizations and 5000 iterations per batch. For multiple comparisons, significance levels were adjusted by sequential Bonferroni corrections (Holm 1979). Deviations from linkage disequilibrium (*L*_D_) between all pairs of loci for each sample were tested using arlequin. The global genetic differentiation (global *F*_ST_) of all gangfisch and the one locus pairwise estimates (*F*_ST_′s) between fish caught at different depths were calculated using genepop. We calculated the pairwise genetic differentiation (*F*_ST_) between fish caught at different depths with 10 000 permutations using arlequin. For comparison of genetic with morphological data, the difference between individuals based on allele frequencies was calculated with factorial correspondence analysis (FCA) using the software genetix version 4.03 (Belkhir et al. 1997).

### Relationship between morphological variation and genetic differentiation

To explore the relationship between variation in body morphology and genetic differentiation on an individual level, we performed two-block partial least squares (2B-PLS) analysis using tpsPLS (part of the TPS software; available for download at http://life.bio.sunysb.edu/morph/index.html (Rohlf and Corti 1993). The 2B-PLS treats two variables symmetrically, thus finding relationships between them without assuming that one is the cause of the other (Rohlf and Corti 1993). The 2B-PLS aims to construct pairs of variables that represent linear combinations between, in our case, the ‘morphology block’ and the ‘genotype block.’ The ‘morphology block’ consisted of all the information of the geometric morphometrics data (landmarks positions analyzed by GPA). Because the ‘genotype block’ contained only the FCA scores as a single variable, we could directly visualize the relationship between the genotype (one variable = FCA score) and the morphology block (one variable distilled from the geometric morphometrics data). As a measure of the relationship, we obtained the correlation coefficient r. As a statistical test for the relationship, we used the software's permutation function that allows to approximate the level of significance of the observed *r*.

## Results

All fish used in our analyses were ripe and running, except for three spent females. Age ranged from 2 to 4 years, the modal age was 3 years. Neither mean age nor sex ratio differed between depths (data not shown).

### Number of gill rakers

Gangfisch spawning at 2 m depth had significantly fewer gill rakers (32.3 ± 4.5 SD) than individuals from 25 (35.2 ± 3.3) and 50 m (37.1 ± 3.4) depth (anova, *F*_(2,87)_ = 12.06; *P* < 0.001; TUKEY *post hoc* test, *P* < 0.05, Fig. S2).

### Growth trajectories

**Figure 3:**
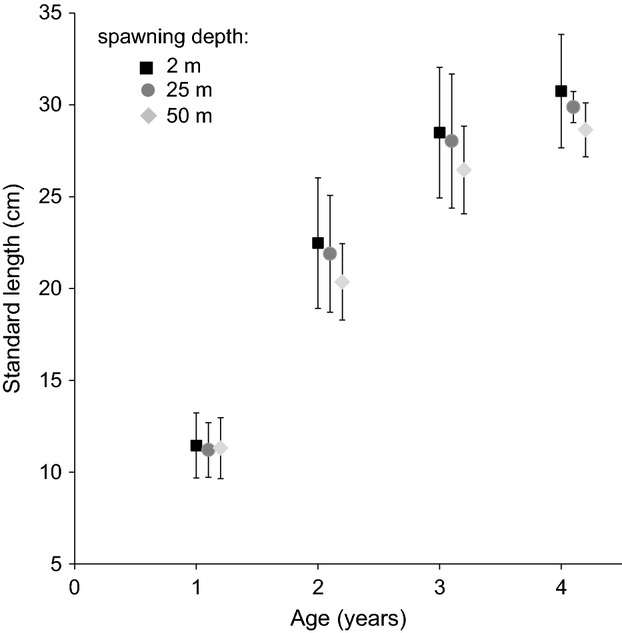
Gangfisch (*Coregonus macrophthalmus*) caught at different depths differ in their growth trajectories. Relationship between standard length and age of gangfisch caught at different depths. Data points depict the mean value of all individuals' back-calculated lengths at ages 1–4 as determined by scalometry (see Methods section for details). Error bars denote standard deviations. Note that the data points were jittered along the *x*-axis to reduce overlap.

Sizes of 3-year-old individuals differed significantly between depths (Kruskal–Wallis test, *H*_(82,60)_ = 12.50, *P* < 0.001). Three-year-old individuals caught at 50 m depth had a significantly shorter standard length than fish of the same age from 2 m (multiple comparisons between ranks; mean rank 2 m = 40, mean rank 50 m = 21, *P *< 0.001). Three-year-old fish spawning at 25 m had an intermediate size not significantly different from either 2 or 50 m (multiple comparisons between ranks; mean rank 25 m = 31, all *P *> 0.05). The correlation between age and size was significant for all depths 2 m: *r* = 0.95, *P *= 0.04; 25 m: *r* = 0.95, *P* = 0.04; 50 m: *r* = 0.96, *P* = 0.03, Fig. 3). ancova revealed that depth had a significant influence on the relationship between size and age (*F*_(2,86)_ = 10.37, *P *< 0.001).

### Body morphology

Geometric morphometric analyses showed that gangfisch body shape differed between the spawning depths (mancova, Wilks' Lambda_(depth)_ = 0.16; *F*_(56,118)_ = 3.14; *P* < 0.001). Body length as a covariable, however, did not affect the differences in morphology between depths (Wilks' Lambda_(centroid size)_ = 0.66; *F*_(28,59)_ = 1.04; *P *= 0.431).

**Figure 4:**
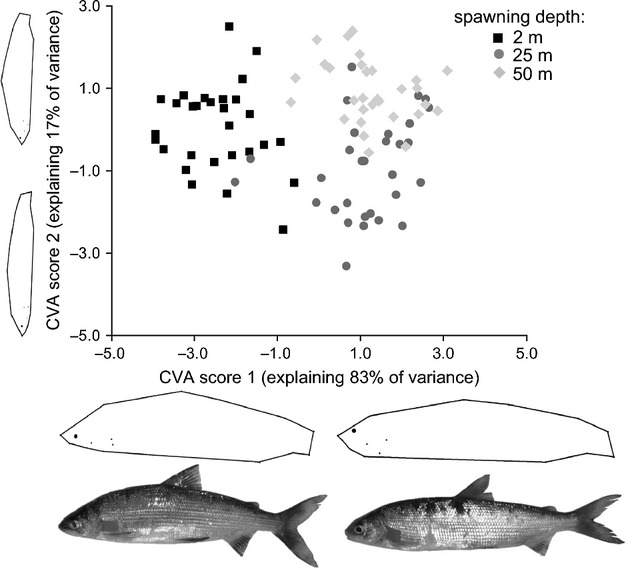
Gangfisch (*Coregonus macrophthalmus*) caught at different depths differ in their body shape. Plot of the two canonical scores describing each individual's body shape. The body shape visualizations at each axis show two hypothetical (not actually caught) individuals created by regression of the canonical scores against the geometric morphometrics data to explore the direction of change across depths. The photographs show real individuals that we selected from the data by visual inspection to illustrate the differences in body shapes actually occurring in the wild.

These differences were reflected in the DFA which showed that individuals can be discriminated into different depths based on their morphology (Wilks' Lambda = 0.18; approximately *F*_(56,120)_ = 134.73; *P* < 0.001). A canonical variance analysis (CVA) on the geometric morphometrics data revealed that a single variance function explains 83% of the variation in body morphology and is sufficient to discriminate between the depths (Eigenvalue = 2.96, Canonical *R *= 0.86; Wilk's Lambda = 0.15, df = 56, *P *< 0.001). The second CVA function explained merely 17% of the variation and did not contribute to the morphological discrimination between depths (Eigenvalue = 0.58, Canonical *R *= 0.60, Wilk's Lambda 0.63, df = 27, *P* = 0.16). However, for completeness, we use both scores to fully visualize an individual's morphology (Fig. 4).

### Trophic niche segregation and stable isotope signatures

Fish from all three depths showed a high degree of niche overlap (58% of the standard area ellipses ± standard deviation (SD) of all pairwise overlaps 16% and 78% of the convex hulls ± SD 14%, Table 2, Figs. S3 and S4). Carbon signatures did not differ between depths (anova, *F*_(2,57)_ = 1.14, *P *= 0.32). However, gangfisch spawning at 2 m depths had marginally more depleted δ C^13^ values [frequently considered indicative of a more littoral diet (Quevedo et al. 2003; Bartels et al. 2012)] than fish spawning at greater depths (Fig. S3). Nitrogen signatures of fish from 50 m depth were significantly higher than those from 25 m (anova, *F*_(2,57)_ = 4.39, *P* = 0.01, Tukey *Post hoc* test: 50 vs 25 m, *P* = 0.01 all other comparisons *P *> 0.1) (Fig. S3).

**Table 2:** Niche segregation of whitefish (*Coregonus macrophthalmus*) caught at three different depths in Lake Constance

	2 m	25 m
25 m	85/41	–
50 m	88/73	61/32

Niche segregation reported as percentage of overlap of trophic niche areas in the form of convex hulls/standard ellipses.

### Trophic niche widths, diversity, and evenness

The analysis of the trophic niches revealed substantial differences in feeding behavior between fish spawning at different depths. The niche width of fish spawning at 2 m was approximately two times larger than the niche width of fish from 25 to 50 m (Table 3, Fig. S3, S4), as indicated by both measures of total niche width [Total convex hull area (TA) and standard error ellipse area (SEA)]. The degree of individual diet specialization was higher in fish spawning at 2 m. This was evident from a larger scatter of stable isotope signatures [distance from centroid (CD)] of gangfisch spawning at 2 m and a higher distance between any individual's isotope signature from 2 m relative to the other individuals' signatures at 2 m depth (lower mean nearest neighbor distance MNND-1) (Table 3, Fig. S3, S4).

**Table 3:** Trophic niche metrics of whitefish (*Coregonus macrophthalmus*) caught at three different depths in Lake Constance

Sampling depth (m)	Niche width (TA)	Niche width (SEA)	Trophic diversity (CD)	Trophic evenness (MNND-1)
2	4.94	1.20	0.74	0.74
25	1.94	0.58	0.68	0.84
50	3.30	0.83	0.64	0.81

Measures of niche widths: TA, total area of convex hull; SEA, standard error ellipse area. Measures of trophic diversity (CD = mean distance to centroid) and trophic evenness (MNND -1 = mean nearest neighbor distance).

### Genetic diversity and differentiation

No null alleles were seen, and no significant deviations from Hardy–Weinberg equilibrium were found at any of the microsatellite loci or in any sample caught at different depths (Table S1). All inbreeding coefficient values (*F*_IS_) were close to zero and not significant. The tests for deviations from linkage disequilibrium were significant in 3 of 90 comparisons at *P *< 0.01; however, because the significant linkage tests involved different pairs of loci in different samples, we concluded that they were more likely effects of type I errors than physical linkage between loci. The global genetic differentiation (*F*_ST_) between gangfisch was 0.003 (Table S1). A small but significant genetic differentiation between gangfisch from 2 and 50 m depth in the pairwise fixation index (*F*_ST_) comparisons was found on the 5% significance level (that, however, disappeared after conservative Bonferroni correction, new significance level 2%), whereas fish from 25 m depth were not significantly different from fish from either 2 or 50 m (Table 4). Pairwise locus by locus comparisons of genetic differentiation showed that population structure was largely attributable to higher *F*_ST_ values at various loci for comparisons of fish caught at 2 m depth vs 50 m depth than for the other two comparisons (Table S2).

**Table 4:** Genetic differentiation between whitefish (*Coregonus macrophthalmus*) caught at three different depths in Lake Constance

	2 m	25 m	50 m
2 m	–	0.242	0.048^*^
25 m	0.002	–	0.698
50 m	0.008^*^	−0.003	–

Given are the pairwise *F*_ST_ values based on 12 microsatellite loci below the diagonal and the corresponding *P*-values above the diagonal, asterisks indicate significant values at *P* < 0.05.

### Relationship between morphological variation and genetic differentiation

**Figure 5:**
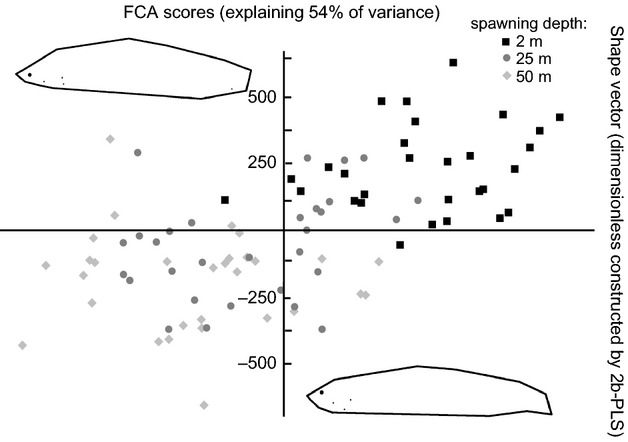
Morphological variation of gangfisch (*Coregonus macrophthalmus*) correlates with genetic differentiation across depths. Note that the abscissa denotes a dimension-less variable or shape vector constructed by 2B-PLS analysis of the geometric morphometric describing the variation in body shape among individual gangfisch. The ordinate denotes the scores of a factorial correspondence analysis (FCA) based on the microsatellites that describe the genetic separation among the depths. The body shape visualizations at each axis show two hypothetical (not actually caught) individuals created by regression of the factorial correspondence scores against the geometric morphometrics data to explore the direction of change across depths.

There was a significant relationship between morphological variation and the genetic differentiation (*r* = 0.58, Fig. 5). The random permutation test found the *r*-value to be greater or as great as 0.58 in only one of 1000 cases suggesting *P* < 0.001.

## Discussion

We investigated the diversification within a single whitefish species caught at different depths and found a subtle degree of phenotypic and genetic diversification between benthic and pelagic specialized individuals.

### Subtle but consistent littoral–pelagic divergence within local gangfisch

The number of gill rakers and growth trajectories are considered highly heritable traits that determine the feeding efficiency in whitefish (Ostbye et al. 1978; Kahilainen et al. 2011). Differences in these traits reflect differential resource use (Kahilainen and Ostbye 2006; Vonlanthen et al. 2004). Individuals with fewer gill rakers grow faster if they forage over littoral bottoms because benthic organisms provide an energy-rich prey source compared with zooplankton (Kahilainen et al. 2003; Kahilainen et al. 2011). High numbers of gill rakers in pelagic whitefish increase small particle retention and thus feeding efficiency on small pelagic food items. Feeding on zooplankton, however, leads to slower growth due to widely dispersed and energy-poor zooplankton (Kahilainen et al. 2003, 2011). The divergence of gangfisch into littoral- and pelagic-feeding phenotypes is further supported by the differences in body morphology among individuals from different depths. In fish, a deeper body allows for higher maneuverability and smaller turning radius and, together with a subterminal snout, increases foraging efficiency on bottom living prey. A more streamlined body and terminal snout in contrast is better adept at prolonged swimming in the pelagic zone searching and consuming widely dispersed food (Pettersson and Hedenström 1997; Svanbäck and Eklöv 2003; Langerhans et al. 2003). Based on the visualizations of the geometric morphometrics analyses, gangfisch from 2 m displayed a more pronounced ‘bottom-feeding-adapted’ body shape as depicted by the ventrally located eye (landmark 16, Fig. S1), the larger gape (landmarks 1–3, Fig. S1), and the deeper body (landmarks 12–15 Fig. S1; visualizations and photographs Fig. 4). Gangfisch from 25 to 50 m depth in contrast had a more ‘pelagic-feeding-adapted’ body shape as depicted by the dorsally located eye, the smaller terminal gape and the slender body (landmarks 16, 1–3, 12–15 Fig. S1; visualizations and photographs Fig. 4).

We also found pronounced differences in niche width and individual diet specialization (trophic diversity and evenness) between fish spawning at shallow and deeper depths. These differences in trophic metrics indicate that individuals spawning at different depths vary in their foraging behavior outside the spawning season. A more diverse food basis of benthic resources (several feeding guilds and phyla of invertebrates) facilitates diet specialization in littoral consumers (Karatayev et al. 2005; Quevedo et al. 2003). This likely underlies the higher trophic diversity of isotopic signatures in gangfisch spawning at 2 m. Pelagic consumers in contrast have fewer opportunities for diet specialization due to a functionally less diverse pelagic food basis consisting of a zooplankton community with fewer feeding guilds chiefly phytoplankton grazers (Vander Zanden et al. 2008; Karatayev et al. 2005). This could explain why deeper water spawning gangfisch showed a lower degree of trophic diversity. The high overlap in isotopic niches between fish spawning at different depths suggests that the subtle phenotypic divergence need not necessarily be reflected in a clear niche separation, at least if niche metrics integrate feeding behavior over several years and if isotopic separation of prey items between habitats remains unknown.

### Evolutionary implications

Even subtle differences in feeding behavior can have bearings on the genetic diversification within a population such that reproductive isolation can arise as a by-product of ecological diversification (Schluter 1996). The process of by-product speciation is considered essential for the formation and maintenance of diversification among species. For example, if males and females mate at times or at places that are related to their feeding behavior, then reproductive isolation can emerge as a by-product of diet specialization (Snorrason et al. 1997; Smith and Skúlason 2010). Our genetic analysis indicates some degree of reproductive isolation as we found small differences in pairwise *F*_ST_ values between gangfisch spawning at 2 and 50 m depth. Even such low levels of genetic differentiation can be ecologically relevant and can reflect a stable diversification of spawning and feeding behavior within a population (Knutsen et al. 2011). The correlation between genetic and phenotypic diversification along the spawning depths indirectly suggests that choice of spawning depth in gangfisch is somewhat connected to the feeding behavior.

Studies on the origins of diversity have almost exclusively focused on retrospective analyses of reproductive isolation between clearly diverged species (Renaut and Bernatchez 1995). A large proportion of the global biodiversity, however, consists of forms or ecotypes of species not fully reproductively isolated (Seehausen 2000). This diversity is maintained by a delicate balance between disruptive selection pushing toward further genetic divergence and gene flow between the divergent forms, pushing toward a phenotypic and genetic homogenization (Seehausen 2000). Changes in the environment can tilt this delicate balance between divergence and homogenization. The eutrophication of a lake has been implicated in the flattening of adaptive peaks which are necessary for disruptive selection to favor extreme phenotypes (Seehausen et al. 2006; Vonlanthen et al. 1999) (Fig. 1). Like many of the world's lakes, however, also Lake Constance underwent re-oligotrophication (Fig. 2B) (IGKB 2011; Vonlanthen et al. 1999). The pre-eutrophication levels of, for example, a low zooplankton biomass have almost been restored (Fig. 2B). This clearly raises the question whether whitefish have the potential to rediverge (Turner 1999; McKinnon and Taylor 2011).

### The future of whitefish diversity in restored Lake Constance

In our study, the gill raker numbers found in gangfisch cover a range between 23 and 42, which is a substantially broader range than before the eutrophication period 33–49: (Nümann 2012; Vonlanthen et al. 1999). Remarkably enough, the range seems to have expanded toward the lower end of the gill raker numbers. This is noteworthy because a low number of gill rakers used to be characteristic of the benthic species that have been lost (*C. gutturosus*, gill raker range 17–25) or declined (*C. arenicolus*, gill raker range 25–29) in Lake Constance and other lakes (Nümann 2012; Vonlanthen et al. 1999).

One possible scenario is that the diversity of whitefish species will re-emerge and shift toward the lower end of gill raker numbers, from which it was lost during eutrophication. This complies with our conceptual model (Fig. 1) that indicates how the restoration of the lake's original resource distribution should re-instate disruptive selection acting to favor specialized phenotypes at the littoral and pelagic extremes of the phenotype distribution. The future scenario of a further divergence in the lake becomes also plausible when considering the genetic data of whitefish in the lake. The introgression of benthic whitefish into gangfisch in Lake Constance likely underlies the higher neutral genetic variation (allelic richness at microsatellite loci) that has been found within some gangfisch from the lake (Vonlanthen et al. 1999). Thus, the shift of the gill raker numbers to the lower end of the distribution we observed in gangfisch might be indicative of both a phenotypic variation and a genetic variation in a population that contains hybrids of previously more clearly separated benthic and pelagic forms. Evolutionary theory predicts that such a source of heritable variation can onset a rapid genetic divergence if natural selection favors extreme phenotypes as, for example, very high or low gill raker numbers (Stockwell et al. 1950).

### Phenotypic plasticity in whitefish divergence

Phenotypic plasticity is important for creating the visible phenotypes selection can target because natural selection is essentially blind toward the genotype (West-Eberhard 2003). When environmental conditions change, a plastic change in the phenotype can lead to a phenotypic accommodation of new traits and eventually further genetic divergence (West-Eberhard 1950). Moderate plasticity, that is, a composite phenotype consisting of plastic and nonplastic traits is optimal for facilitating such rapid adaptations (Price et al. 2000). Recent experimental work confirms that divergence of whitefish species is facilitated by a combination of plastic and heritable traits. Common garden experiments demonstrated that both variation in plastic feeding behavior and nonplastic gill raker number drives the divergence into benthic and pelagic whitefish species (Lundsgaard-Hansen et al. 1995). The divergence in feeding behavior and body morphology we found in gangfisch might result from phenotypic plasticity. The divergence in heritable traits such as gill rakers and growth rates suggests a combination of plastic and heritable traits. The phenotypic plasticity in feeding-related traits could bring individuals into the realm of attraction of newly formed adaptive peaks in the lake (*cf*. Fig. 1). A congruent variation in heritable traits can then complement the process toward rapid adaptation (Agrawal 2001; Price et al. 2000).

### Conservation of whitefish diversity

Studies in other polymorphic fish advocated the protection of the different forms because they contain a genetic diversity and thus ecosystem function that is worth conserving (Etheridge et al. 2010a). Such evolutionary significant units (*in sensu* Fraser and Bernatchez 2001) are commonly described in salmonid fish; for example, the different populations of Atlantic salmon (*Salmo salar*) isolated among different spawning rivers. More relevant for the evolutionary application of this concept in our case is Waples' (2012) account for evolutionary significant units as representing ‘a product of past evolutionary events that represents the reservoir upon which future evolutionary potential depends.' Along these lines, the diversity we detected in gangfisch could represent the reservoir of variation for future diversification into, once again, several species in the lake. The role of phenotypic plasticity should also be appreciated for the development of whitefish diversity in the lake's future. Phenotypic plasticity itself has a heritable component that can evolve on contemporary timescales (Lind and Johansson 2007). Phenotypic plasticity can also be lost within contemporary timescales when a population's ability to respond plastically is selected against by humans through, for example, artificial breeding (Collyer et al. 2011). This can impair a population's potential to adapt to environmental change. For example, plasticity in gene expression in response to environmental changes was found to be lower in domesticated than in wild Atlantic salmon (Debes et al. 2012). This underscores the need for the conservation of the local gangfisch. The potential for contemporary evolution would be secured by supporting the current state of divergence hence allowing individuals to adapt to ongoing and future changes in the adaptive landscape (Hendry et al. 2011).

### Implications for fisheries management

To preserve or at least not to endanger the potential for divergence in gangfisch, the fisheries management and stocking practices in the lake should be adapted. There is no detailed information on the extent to which gangfisch stocking contributes to the lake's gangfisch population, but stocking is conducted for both gangfisch and blaufelchen. In one year, the blaufelchen stocking material contributed to as much as 83% of the total population in the lake (Eckmann 2012). The stocking material for the hatcheries is obtained by a catch of spawners of both gangfisch and blaufelchen. Spawning location of gangfisch does not overlap with blaufelchen, and they are each bred separately in the hatcheries guaranteeing a species-specific stocking (Eckmann 2012). Our findings of depth-specific divergence in gangfisch imply that the catch depths of spawners should greatly affect the genetic and phenotypic identity of the stocking material produced. At present, the catch of gangfisch for production of fry is concentrated around depths of approximately 20 m (information from local fishermen). Our analyses show that the spawners at 25 m depth were most indifferent from gangfish caught at the other two depths, whereas individuals from 2 vs 50 m differed most. If fry is produced from spawners from only 20 m as a single depth, a particular genetic or phenotypic variety of gangfisch may be artificially supported. We therefore propose that the catch depths for fry production be at least extended to comprise deeper (50 m) and shallower (2 m) depths. This could minimize the stocking bias currently taking place.

A strictly depth-specific catch and subsequent separated hatching of gangfisch fry would best contribute to protect and enhance the integrity of different evolutionary significant units. But this would also entail higher costs for hatcheries and a higher fishing effort for fishermen as various catch depths then cannot be chosen freely to maximize yield. Which incentives could motivate stakeholders to support the conservation of whitefish biodiversity? One incentive could be the revival of the fisheries for gangfisch in shallow waters. Fishing gangfisch in shallow lake areas was a century-old tradition in Lake Constance with considerable sociocultural and economic value (Fig. S5). Coinciding with the onset of the eutrophication in the late 1960s and the loss of whitefish from shallow waters, this tradition was abandoned. Supporting the redivergence in gangfisch might eventually bring back a plentiful catch at shallow depths. This would allow for a revival of this cultural heritage adding socioeconomic value to the whitefish biodiversity thus motivating stakeholder involvement (Addis et al. 2012).

## Conclusions

Many lakes across the globe and especially those that historically featured polymorphic forms of freshwater fish have been re-oligotrophied (Anderson et al. 2005; Vonlanthen et al. 1999). Our data provide an example of how subtle diversity within a local population could secure a species' potential for future evolutionary change. Future work on genetic and phenotypic diversity within populations of species in other anthropogenically affected ecosystems will help to elucidate how restoration toward pristine environmental conditions and a sustainable management can bring back biodiversity. The socioeconomic value of whitefish could play an important role in motivating stakeholder involvement in the process of conservation.

## Data archiving statement

Data for this study are available at the Dryad repository: doi:10.5061/dryad.m9s46.

## Supporting Information

Figure S1 Schematic drawing of a whitefish and location of the 16 landmarks used for the geometric morphometrics analyses.

Figure S2 Number of gill rakers of gangfish caught at different depths.

Figure S3 δC13 and δN15 biplot of gangfisch, total niche area as convex hull (TA) (*Coregonus macrophthalmus*).

Figure S4 δC13 and δN15 biplot of gangfisch, total niche area as standard ellipses (SEA) (*Coregonus macrophthalmus*).

Figure S5 Traditional catch of gangfisch (*Coregonus macrophtalamus*) in shallow waters of Lake Constance.

Table S1 Population genetic analysis summary statistics for whitefish (*Coregonus macrophthalmus*).

Table S2 Single locus pairwise comparisons of genetic differentiation (F_ST_) between whitefisch (*Coregonus macrophthalmus*) caught at three different depths in Lake Constance.
