# Supplementary material for: Phenotypic and genetic divergence within a single whitefish form – detecting the potential for future divergence
Source: Evol Appl. 2013 Sep 10;6(8):1119–32. doi: 10.1111/eva.12087 (PMC3901543; doi:10.1111/eva.12087)
Supplement: Figure S1 — Schematic drawing of a whitefish and location of the 16 landmarks used for the geometric morphometrics analyses. [file eva0006-1119-sd1.pdf]

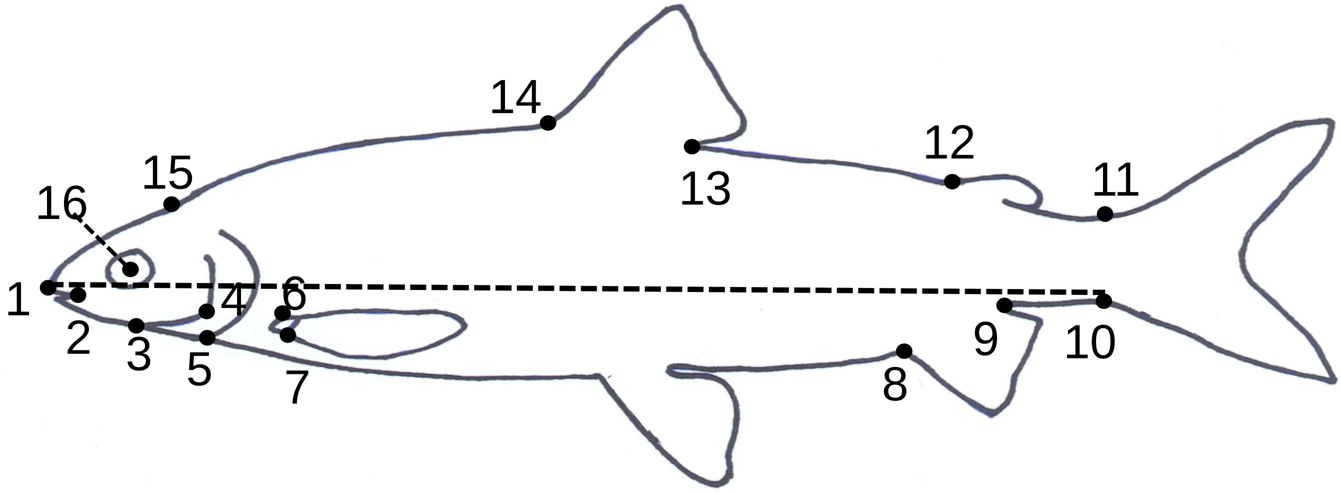

Fig. S1: Schematic drawing of a whitefish and location of the 16 landmarks used for the geometric morphometrics analyses. Note that landmark 4 is a sliding (semi-) landmark. The dotted line represent the axis along which possibly present bending or arching of the fish body was removed prior to any further analysis of the data. See materials and methods in the main text for more information. Description of landmark positions:

1: Utmost part of the snout at proximal end of upper lip
2: Endpoint of the mouth opening at distal end of upper lip
3: Point where the preoperculum and the operculum meet
4: Ventral end of operculum
6: Dorsal insertion of the pectoral fin
7: Ventral insertion of the pectoral fin
8: Proximal insertion of anal fin
9: Distal insertion of anal fin
10: Ventral insertion of the caudal fin
11: Distal insertion of the caudal fin
12: Proximal insertion of the adipose fin
13: Distal insertion of the dorsal
14: Proximal insertion of the dorsal fin
15: Emargination where different parts of scull come together and where scales begin.
16: Middle point of the eye
