# Supplementary material for: Phenotypic and genetic divergence within a single whitefish form – detecting the potential for future divergence
Source: Evol Appl. 2013 Sep 10;6(8):1119–32. doi: 10.1111/eva.12087 (PMC3901543; doi:10.1111/eva.12087)
Supplement: Figure S2 — Number of gill rakers of gangfish caught at different depths. [file eva0006-1119-sd2.pdf]

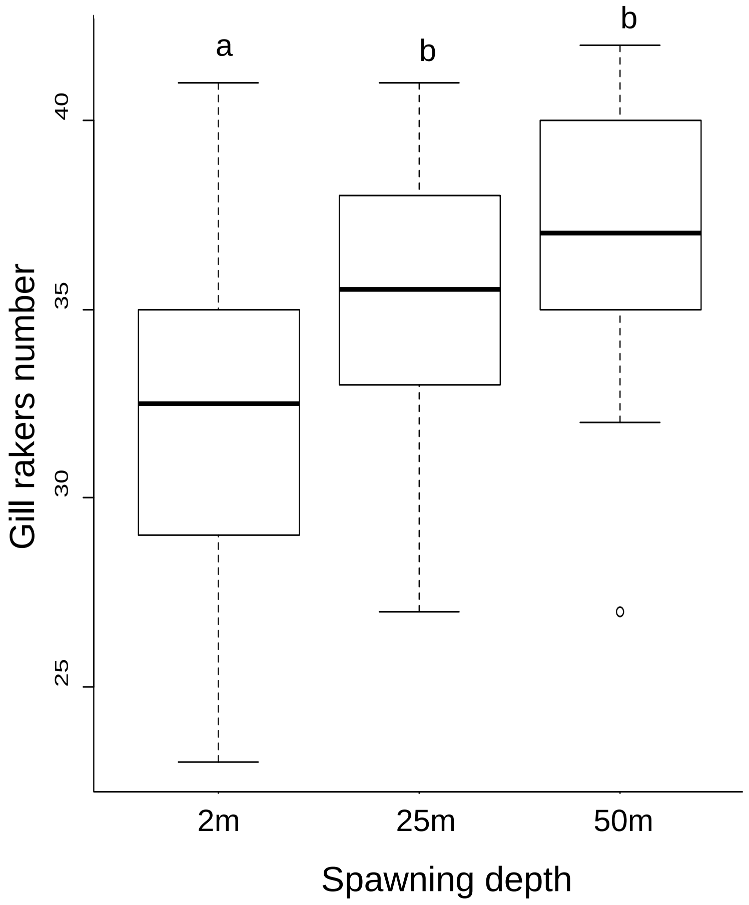

Fig.S2: Gangfisch (*Coregonus macrophthalmus*) caught at different depths differ in the number of gill rakers. Box whisker plots of the number of gill rakers on the first right gill arch (each depth's n=30). Boxes depict the 25% and 75% percentiles. Errors bars depict the non-outlier range, lines the median, and circles outliers. Letters indicate statistical differences after Tukey post-hoc tests.
