# Supplementary material for: Phenotypic and genetic divergence within a single whitefish form – detecting the potential for future divergence
Source: Evol Appl. 2013 Sep 10;6(8):1119–32. doi: 10.1111/eva.12087 (PMC3901543; doi:10.1111/eva.12087)
Supplement: Figure S3 — δC13 and δN15 biplot of gangfisch, total niche area as convex hull (TA) (Coregonus macrophthalmus). [file eva0006-1119-sd3.pdf]

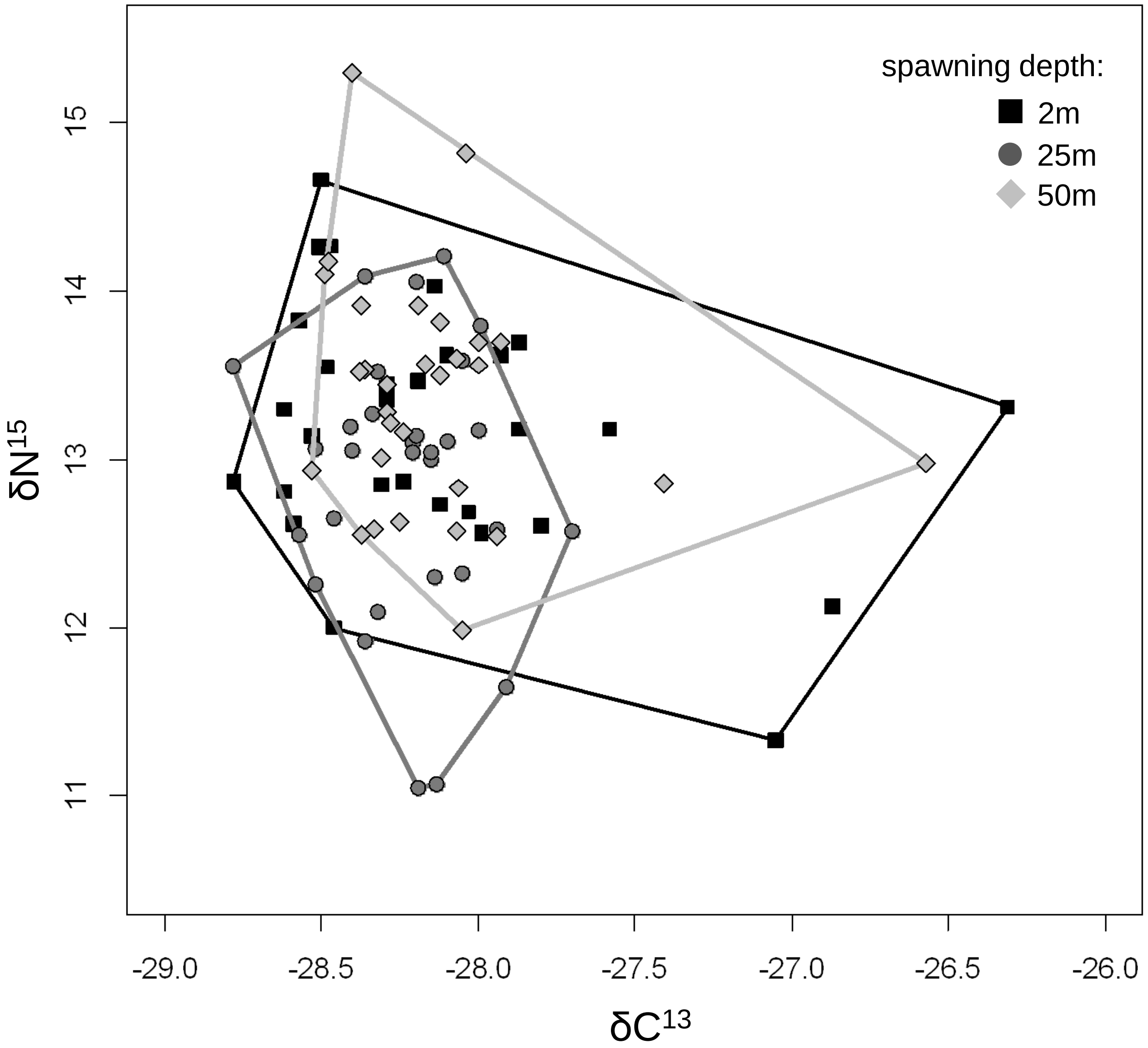

Fig. S3: δC^{13} and δN^{15} biplot of gangfisch (*Coregonus macrophthalmus*). The polygons depict the total niche area as convex hull (TA) for all individuals caught spawning at each depth.
