# Supplementary material for: Phenotypic and genetic divergence within a single whitefish form – detecting the potential for future divergence
Source: Evol Appl. 2013 Sep 10;6(8):1119–32. doi: 10.1111/eva.12087 (PMC3901543; doi:10.1111/eva.12087)
Supplement: Figure S4 — δC13 and δN15 biplot of gangfisch, total niche area as standard ellipses (SEA) (Coregonus macrophthalmus). [file eva0006-1119-sd4.pdf]

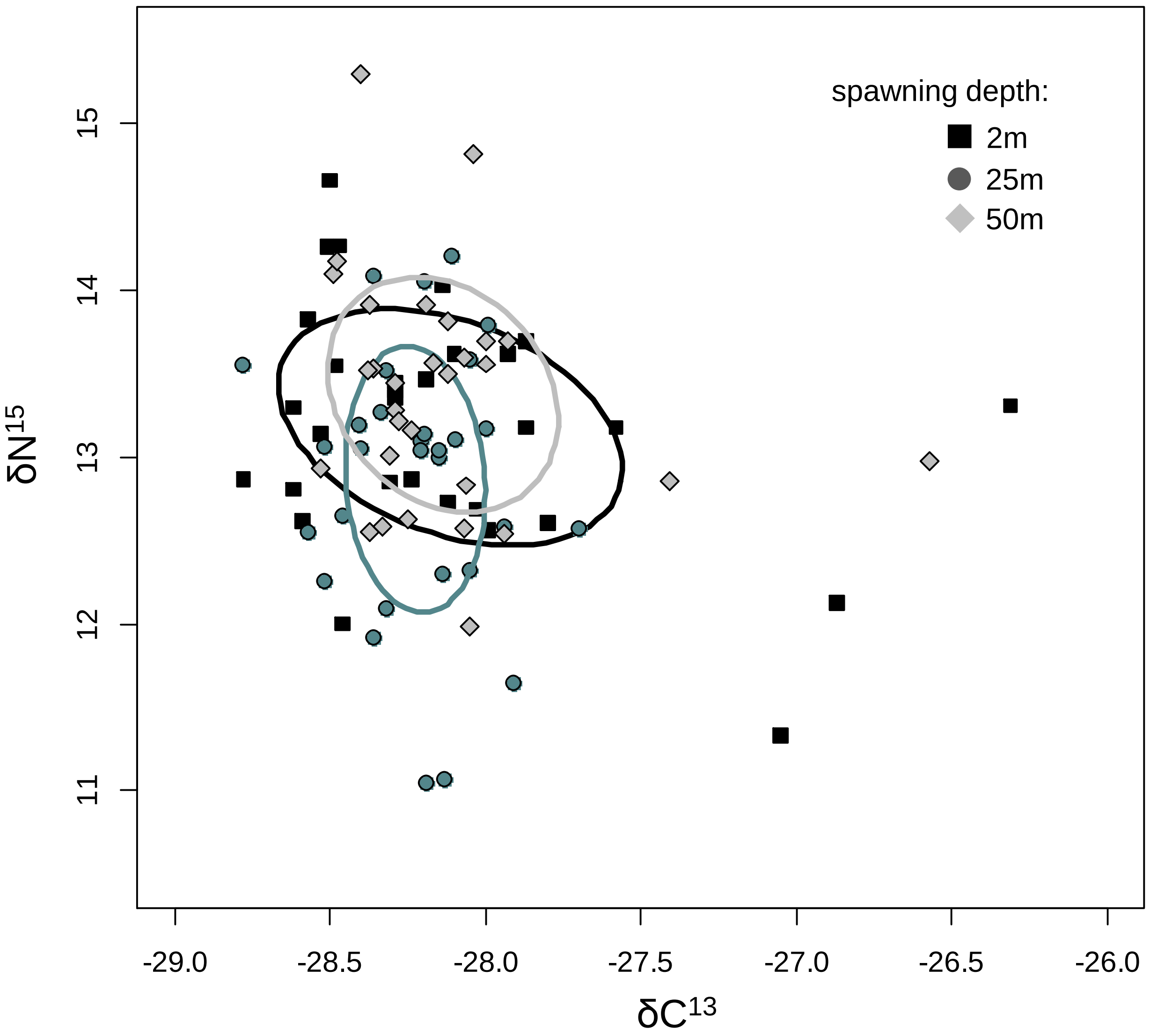

Fig. S4: δC^{13} and δN^{15} biplot of gangfisch (*Coregonus macrophthalmus*). The total niche areas are depicted as standard area ellipses (SEA) for all individuals caught spawning at each depth.
