# Supplementary material for: Phenotypic and genetic divergence within a single whitefish form – detecting the potential for future divergence
Source: Evol Appl. 2013 Sep 10;6(8):1119–32. doi: 10.1111/eva.12087 (PMC3901543; doi:10.1111/eva.12087)
Supplement: Figure S5 — Traditional catch of gangfisch (Coregonus macrophtalamus) in shallow waters of Lake Constance. [file eva0006-1119-sd5.pdf]

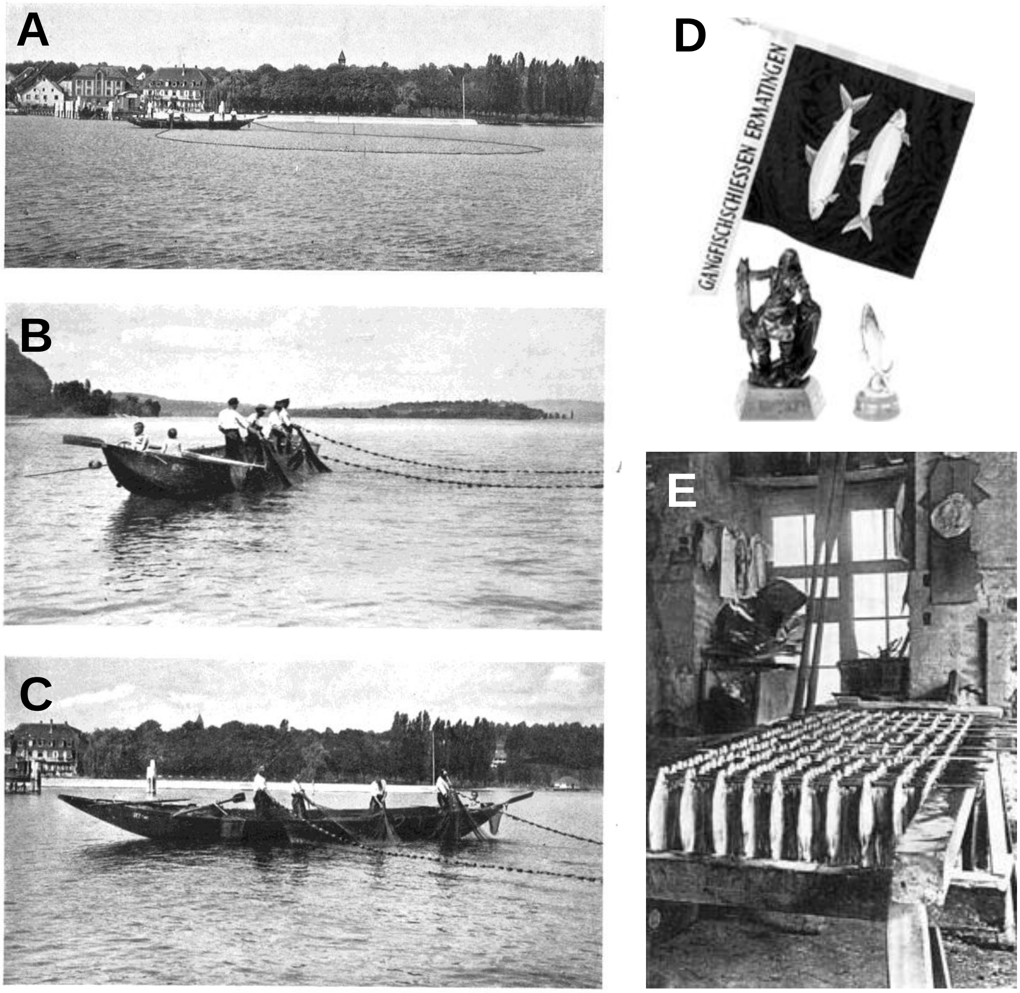

Fig. S5: Traditional catch of gangfisch (*Coregonus macrophtalamus*) in shallow waters of Lake Constance. A-C, Sequence of events from top to bottom: continued hauling in of the seine-like net by fishermen standing in the boat. The photographs were taken in approx. 1 km distance from the catch location of gangfisch in this study.

The boats used for catching gangfisch are a specifically designed 15 m long vessel named gangfisch-segi that had a short distance between keel and waterline and space for up to 18 fishermen. Fishing for gangfisch was conducted all-year round (with fewer fishermen, see photographs A-C) but had its peak season during spawning time of gangfisch because females containing eggs were considered a delicacy and spawners assembling in the shallow-most littoral areas were easiest to catch. For catching gangfisch during spawning season fishermen assembled a large seine-like net by a joint effort. Each fisherman would bring a piece of seine and prior to the spawning season up to 18 fishermen's seine pieces would be nit together to a single large seine following a detailed plan. The fishermen would then build a single crew of 18 for the catch of spawners in the shallow depths close to the shore. No detailed records of historical catches exist but some sources speak of as much as 46,000 spawning gangfisch during one December in 1534. The joining of fishermen families and the catch was celebrated with a gangfisch feast that is likely to have played a significant role for the social cohesion of the local communities. Only one not fish-related feature of this feast has survived up to the present day. The Gangfischschiessen, a sports-shooting competition attracts up to 2500 visitors annually to a small fishermen village that used to be famous for its gangfisch feasts. Historically, gangfisch were the prize for the best shooter (Fig.S5 D). Today the last remaining boats from that era are planned to be the central part of a new museum which further highlights the role such traditions have been playing and can continue to play for the identity of communities around the lake. Also the economic importance of the gangfisch fisheries was substantial as exemplified by the historical export of catches. Smoked gangfisch (Fig. S5 E) was a delicacy that was sold to markets as far away as Italy and France (both several hundred kilometers away). The last documented catch of gangfisch spawners with a communal gangfisch-segi dates back to 1967.

(Photographs' credits: A-C: unknown photographer, taken from the book "Handbuch der Binnenfischerei Mitteleuropas, ed: Demoll R. and Maier H.N., Schweizerbart sche Verlangsbuchhandlung (Nägele u. Obermiller)," 1941. B: courtesy of Rene Weber, president of the local Gangfischschiessen organizing committee of Ermatingen, Switzerland. C: courtesy of the association for a Lake-Constance fisheries-museum in Ermatingen (Verein für ein Bodensee-Fischereimuseum in Ermatingen).

Further references: http://www.kulinarischeserbe.ch; Tagblatt Online, 27. August 2012 01:35:34; Hüster Plogmann H (2006) Forschungen in Augst, Vol.39,; Oken L (1836) Allgemeine Naturgeschichte für alle Stände. vol. 6 = Thierreich, Dritter Bandpp366, Stuttgart: Hoffmann'sche Verlags-Buchhandlung; Deutsches Wörterbuch von Jacob Grimm und Wilhelm Grimm 1854-1961, Vol 4, p. 1248. All online sources accessed: 05/13/2013)
