# Supplementary material for: Phenotypic and genetic divergence within a single whitefish form – detecting the potential for future divergence
Source: Evol Appl. 2013 Sep 10;6(8):1119–32. doi: 10.1111/eva.12087 (PMC3901543; doi:10.1111/eva.12087)
Supplement: Table S1 — Population genetic analysis summary statistics for whitefish (Coregonus macrophthalmus). [file eva0006-1119-sd6.docx]

**Table T1:** Population genetic analysis summary statistics for whitefish (*Coregonus macrophthalmus*). Given is the global genetic differentiation (F_ST_), the observed (H_O_) and expected (H_E_) heterozygosity with the corresponding P-value of Fisher´s exact tests for deviation from the Hardy-Weinberg equilibrium, and the number of loci that deviated from linkage equilibrium (N_LD_)

| **spawning depth (m)** | **N** | **Global F_ST_** | **H_O_** | **H_E_** | **p-value** | **N_LD_ p<0.01** |
| --- | --- | --- | --- | --- | --- | --- |
| **2** | 30 |  | 0.62 | 0.59 | 0.35 | 2 |
| **25** | 30 | 0.003 | 0.56 | 0.56 | 0.44 | 0 |
| **50** | 30 |  | 0.55 | 0.57 | 0.85 | 1 |
