# Supplementary material for: Phenotypic and genetic divergence within a single whitefish form – detecting the potential for future divergence
Source: Evol Appl. 2013 Sep 10;6(8):1119–32. doi: 10.1111/eva.12087 (PMC3901543; doi:10.1111/eva.12087)
Supplement: Table S2 — Single locus pairwise comparisons of genetic differentiation (FST) between whitefisch (Coregonus macrophthalmus) caught at three different depths in Lake Constance. [file eva0006-1119-sd7.docx]

**Table T2:** Single locus pairwise comparisons of genetic differentiation (F_ST_) between whitefisch (*Coregonus macrophthalmus*) caught at three different depths in Lake Constance

| **Locus** | **2m vs. 25m** | **25m vs. 50m** | **2m vs. 50m** |
| --- | --- | --- | --- |
| **BWF1** | -0.0138 | -0.0092 | -0.0125 |
| **BWF2** | 0.0165 | -0.0122 | 0.0186 |
| **CoclLav18** | 0.0147 | -0.0066 | 0.0302 |
| **CoclLav4** | -0.0057 | -0.0146 | 0.0002 |
| **CoclLav45** | 0.0548 | 0.0391 | -0.0136 |
| **Cisco157** | 0.0008 | -0.0066 | 0.0354 |
| **CoclLav10** | 0.0049 | -0.0080 | 0.0136 |
| **CoclLav49a** | 0.0135 | 0.0176 | 0.0476 |
| **CoclLav6** | -0.0140 | -0.0071 | -0.0069 |
| **CoclLav61** | -0.0159 | 0.0104 | 0.0055 |
| **CoclLav68** | 0.0111 | -0.0031 | -0.0091 |
| **SsBglIIM26self** | -0.0109 | -0.0038 | -0.0096 |
| **All loci** | **0.0027** | **-0.0025** | **0.0084** |
